# Clinical presentation and maternal-fetal outcomes of Mirror Syndrome: A case series of 10 affected pregnancies

**DOI:** 10.1177/1753495X211058043

**Published:** 2021-11-29

**Authors:** Hussain Mogharbel, Jennifer Hunt, Rohan D’Souza, Sebastian R Hobson

**Affiliations:** 1 Division of Maternal-Fetal medicine, Department of Obstetrics and gynecology, 7938 University of Toronto, Toronto, ON, Canada; 2 Division of Maternal-Fetal medicine, Department of Obstetrics and gynecology, Max Rady College of Medicine, 8664 University of Manitoba, Winnipeg, MB, Canada; 3 Department of Obstetrics and gynecology, King Abdulaziz university, Jeddah, KSA

**Keywords:** Mirror Syndrome, Ballantyne syndrome, placental edema, triple edema, maternal hydrops syndrome

## Abstract

**Background:**

Mirror Syndrome, also known as Ballantyne syndrome, is a rare condition with fewer than 120 cases described in the literature. A simultaneous edematous state of the mother, fetus and placenta is pathognomonic, with the maternal condition frequently presenting with signs and symptoms similar to that of preeclampsia.

**Objective:**

Our aim was to add to the international body of literature through identification of all cases of Mirror Syndrome at two Canadian tertiary obstetric centres and characterize the maternal presentation, laboratory findings, and perinatal outcomes.

**Methodology:**

We performed a retrospective chart review of all cases of fetal hydrops from two tertiary centres in Winnipeg (Manitoba, Canada) between 2000 and 2019. There were 276 cases of fetal hydrops during this period, of which 10 cases satisfied the diagnostic criteria for Mirror Syndrome where maternal and perinatal outcomes were analysed.

**Results:**

The median gestational age at diagnosis with Mirror Syndrome was 23 weeks and 3 days of gestation and at birth was 25 weeks and 0 days of gestation. The majority of women were multiparous (80%) and had elevated maternal body mass index (median 33 kg/m^2^). The most common maternal clinical findings included weight gain (100%) and hypertension (90%). The most common laboratory findings included low hematocrit (100%), hypoalbuminemia (80%), anemia (70%) and hyperuricemia (70%). Structural anomalies were observed in 50% of cases, over half of the fetuses were stillborn (66.7%) and one quarter of pregnancies resulted in neonatal deaths (25%). The median time until maternal improvement of Mirror Syndrome was 2 days postpartum.

**Conclusion:**

Mirror Syndrome affected 3.6% of all cases of fetal hydrops in our cohort, and showed associations with multiparity, elevated BMI, hemodilution, hypoalbuminemia, anemia and hyperuricemia. Delivery is frequently required for fetal and/or maternal indications and symptoms usually improved rapidly after delivery.

## Introduction

Ballantyne syndrome was first described by J.W. Ballantyne in 1892 and is a condition hallmarked by maternal edema, placentomegaly, and fetal hydrops.^1^ Various names have been used to describe the condition of triple edema, including maternal hydrops syndrome and pseudotoxemia, however it was not until 1956 that Mirror Syndrome was given its contemporary name by D.T. O’Driscoll.^2,3^

Prior to the 1970s, Mirror Syndrome was thought to be a consequence of Rhesus isoimmunization.^3^ However, with the advent of ultrasound and advances in prenatal diagnosis, it became clear that multiple etiologies could eventually lead to fetal hydrops and Mirror Syndrome.^4^ While the precise pathophysiology of Mirror Syndrome remains poorly understood, current theories of genesis are akin to that of preeclampsia.^5^ In fact, both disease entities share the common characteristic of widespread endothelial cell dysfunction manifesting as maternal hypertension and peripheral/systemic edema.

Mirror Syndrome remains a very rare, perhaps under-reported, diagnosis with fewer than 120 documented cases in the literature, usually published as sporadic case reports which lack specific clinical features, since the condition usually resolves after fetal treatment, childbirth or fetal death.^6,7^ Furthermore, it is challenging to recognize cases of Mirror Syndrome in women presenting with non-specific signs and symptoms such as peripheral edema, weight gain, dyspnea, hypertension, headache and visual disturbances,^8^ unless concurrent fetal hydrops and placentomegaly is demonstrated on obstetric ultrasound.

The aim of this study was to highlight the associations and clinical outcomes in cases of Mirror Syndrome identified and managed at two Canadian tertiary care centers.

## Methods

Research ethics approval was obtained from the University of Manitoba Ethics Board, Health Science Center and Saint Boniface Hospital Research Committees [HS20668(H2017:119) and RRC/2017/1689, respectively]. We performed a retrospective chart review identifying all cases of fetal hydrops from Health Science Center and Saint Boniface Hospital, Winnipeg, Manitoba, between 2000 and 2019. Fetal hydrops was defined as the accumulation of fluid in two or more fetal compartments, including ascites, pleural effusion, pericardial effusion, and/or skin edema.^1^ There were 276 cases of fetal hydrops identified during this period, of which 10 cases met the criteria for Mirror Syndrome - defined as the presence of placentomegaly on ultrasound, evidence of maternal edema on clinical examination and fetal hydrops (Table 1).^2^

**Table 1.** Mirror Syndrome diagnostic criteria for inclusion.

Finding	Definition
Placentomegaly	Placental thickness > 4 cm up to 20 weeks of gestation and > 6 cm after 21 weeks of gestation
Fetal Hydrops	Accumulation of fluid in two or more fetal compartments, including ascites, pleural effusion, pericardial effusion, and/or skin edema
Maternal edema	Based on clinical examination: peripheral pitting edema, sacral edema, facial edema, pulmonary edema, ascites etc,

## Results

Case characteristics are demonstrated in Table 2. The median maternal age was 30 years (range 24–34 years) and BMI 33 kg/m^2^ (range 20–42 kg/m^2^), with a median parity of 2 (range 0–3). There were two twin gestations within the 10 pregnancies resulting in a total of 12 fetuses. Fetal hydrops was diagnosed at a median age of 19 weeks and 2 days of gestation (range 16 weeks and 3 days to 28 weeks and 5 days), while the diagnosis of Mirror Syndrome was made at a median of 22 weeks and 3 days of gestation (range 16 weeks and 6 days to 31 weeks and 1 day). The median gestation at birth was 25 weeks (range 17 weeks and 3 days to 31 weeks and 6 days). Labour was induced in 6 cases and in 4 cases labour occurred spontaneously. The most frequent indications for labor induction were maternal hypertension and symptomatic maternal volume overload with associated dyspnea. Regarding mode of delivery, 10 fetuses were delivered vaginally, 1 by emergency caesarean section after spontaneous onset of labour at 31weeks and 6 days of gestation for a fetus with Rhesus isoimmunization and 1 required dilation and evacuation for incomplete molar pregnancy. The median interval from diagnosis to delivery was 8 days (range 1−48 days), and improvement of the maternal condition (defined as improvement in signs, symptoms or biochemical markers) was seen after a median of 2 days (range 1−5 days). Most relevant to the diagnosis of Mirror Syndrome was the observation of increased maternal hemodilution at the time of delivery with a median hematocrit of 20% (range 17−22%).

**Table 2.** Case characteristics.

Case	Maternal Age	Maternal BMI	Gravidity	Parity	Maternal Rhesus type	Maternal Antibody	Number of fetuses	GA at diagnosis of fetal hydrops	GA at diagnosis of Mirror Syndrome	GA at birth	Diagnosis to birth (days)	HCT at delivery (%)	Birthweight <10^th^ centile	Days until maternal Improvement
**1**	27	42	3	1	+	−	1	31 + 1	31 + 1	31 + 6	5	20	–	2
**2**	28	40	5	3	+	+ Lewis	1	19 + 0	19 + 0	19 + 1	1	20	+	5
**3**	24	32	3	1	+	-	1	22 + 0	23 + 5	24 + 3	17	21	–	1
**4**	33	32	1	0	+	−	2	20 + 6	20 + 6	21 + 1	2	22	–/NA	2
**5**	30	35	3	1	+	−	1	21 + 6	26 + 5	28 + 2	45	18	–	2
**6**	30	34	5	2	+	−	1	22 + 3	23 + 0	23 + 6	10	19	–	3
**7**	30	20	1	0	–	+ Anti-D	1	29 + 5	29 + 5	31 + 6	15	19	–	3
**8**	31	23	4	1	+	−	2	26 + 3	26 + 3	26 + 4	1	20	–/ +	2
**9**	26	31	2	1	+	−	1	24 + 0	24 + 3	25 + 4	11	21	–	4
**10**	34	36	2	1	+	−	1	16 + 6	16 + 6	17 + 3	4	17	–	2
**Median**	**30**	**33**	**3**	**1**	**N/A**	**N/A**	**1**	**22 + 3**	**24 + 1**	**25 + 0**	**7**	**20**	**N/A**	**2**

BMI = body mass index, GA = Gestational age, HCT = hematocrit.

With regard to the presumed etiologies of fetal hydrops, 50% (5 pregnancies) were associated with fetal structural anomalies including 2 fetuses with sacrococcygeal teratoma, 1 with Congenital Pulmonary Airway Malformation, 1 with hypoplastic left heart syndrome and 1 with cystic hygroma and facial abnormalities. Of the other 5 pregnancies, there was 1 case each of Rhesus isoimmunization, twin-to-twin transfusion syndrome, twin reversed arterial perfusion sequence, incomplete molar pregnancy,coxsackie virus B3 infection.

Maternal findings are listed in Table 3. All women demonstrated hemodilution, and median rapid weight gain of 1800 g (range 800–2500 g) in less than 1 week, with 90% being hypertensive (blood pressure over 140/90 mmHg). Other common observations included hypoalbuminemia defined as serum albumin under 26 g/L (80%), maternal anemia defined as maternal haemoglobin at the time of presentation of below 110 g/L (70%). Five women developed preeclampsia (defined as a combination of hypertension with new-onset proteinuria), and of these, 2 were delivered soon after diagnosis.

**Table 3.** Frequency of maternal findings in the 10 cases of Mirror Syndrome.

Maternal findings	N (%)	Median (range)
Hemodilution (hematocrit < 30%)	10 (100%)	Hematocrit: 20% (17−22%)
Weight gain	10 (100%)	1800 g (800−2500 g)
Hypertension (> 140/90 mmHg)	9 (90%)	Systolic BP 154 mmHg (135−165 mmHg)
		Diastolic BP 93 mmHg (86−110 mmHg)
Hypoalbuminemia (<26 g/L)	8 (80%)	18 g/L (8−29 g/L)
Anemia (Hemoglobin <110 g/L)	7 (70%)	89 g/L (75−117 g/L)
Hyperuricemia (UA >300 umol/L)	7 (70%)	452 umol/L (290−578 umol/L)
Proteinuria (PCR >30 mg/mmol)	5 (50%)	74 mg/mmol (24−287 mg/mmol)
Thrombocytopenia (Plt <150 × 10^9^/L)	5 (50%)	83 (79−167 × 10^9^/L)
Dyspnea	2 (20%)	–

BP = blood pressure, UA = uric acid, PCR = protein creatinine ratio, Plt = platelet.

Table 4 highlights the perinatal outcomes and possible etiologies. The only infant that survived the neonatal period was a case of Rh isoimmunization that required 4 intrauterine blood transfusions prior to delivery and was delivered prematurely with hydrops at 31 weeks and 6 days of gestation by emergency caesarean section after spontaneous onset of labour. An additional case underwent fetal intervention with radiofrequency ablation (RFA) of the acardiac fetus in a case of twin reversed arterial perfusion syndrome.

**Table 4.** Fetal and neonatal outcomes in the 12 infants.

Outcome	N (%)	Etiology
Stillbirth	8 (66.7)	1 viral infection (coxsackie Virus B3)
		1 TRAP (diagnosis at 20 weeks and 6 days of gestation)
		2 Sacrococcygeal teratoma
		1 Cystic hygroma and facial abnormalities.
		1 Incomplete molar pregnancy
		1 TTTS
Neonatal death	3 (25)	1 CPAM
		1 Twin, pregnancy complicated by TRAP and co-twin demise
		1 Hypoplastic left heart syndrome
Living	1 (8.3)	Rh alloimmunization

TRAP = Twin reversed arterial perfusion sequence, TTTS = Twin to Twin transfusion syndrome, CPAM = Congenital pulmonary airway malformation, Rh = Rhesus.

Finally, Table 5 compares maternal findings at the peak of the condition and at discharge, to characterize clinical improvement following delivery.

**Table 5.** Improvement of maternal symptoms.

Symptom/Finding	Highest level (median/range)	At discharge (median/range)
Blood pressure (mm Hg)	154/93 (135-165/75-100)	136/84 (128-148/70-88)
Hemoglobin (g/L)	89 (75−107)	96 (82−112)
Hematocrit (%)	20 (17−22)	28 (26−31)
Serum albumin (g/L)	18 (8−24)	25 (22−28)
Uric acid (umol/L)	452 (306−578)	280 (254−320)

## Discussion & Conclusion

The current study examined the clinical findings and outcomes of 10 pregnancies diagnosed with Mirror Syndrome among 276 cases of fetal hydrops, with a 3.6% incidence in our cohort, and adds to the relatively small body of literature on this rare condition. In our cohort, the diagnosis of Mirror Syndrome was associated with multiparity, elevated BMI, hemodilution, hypoalbuminemia, anemia and hyperuricemia in the setting of fetal hydrops with or without structural anomalies. Labour was induced in 6 cases for maternal indications, and maternal clinical features improved within 5 days postpartum in all instances. Fetal outcomes were poor with only one infant surviving the neonatal period.

With regard to diagnosis, we observed a median gestational age of 22 weeks and 3 days of gestation, which is similar to findings by Allarakia et al., in the most recent systematic review on Mirror Syndrome involving 113 women.^6^ Their group also reported that it was not clinically relevant to perinatal outcomes whether it was the maternal or the fetal features that occurred first in the temporal sequence of events, but rather the final combined diagnosis of Mirror Syndrome and triple edema itself.

We observed a non-immune or non-infectious cause for fetal hydrops in 80% of our cases, which is also consistent with the current literature.^6^ The presiding cause of fetal hydrops in our series was structural anomalies which has also been described and correlates with the poor fetal outcomes observed with high rates of stillbirth and neonatal death, particularly with the added sequelae of prematurity. Allarakia et al. showed an overall survival rate of 32.7%,^6^ whereas in our series of 12 offspring, only 8.3% survived which may be a function of the small sample size and the high proportion of fetuses with hydrops, structural anomalies and extreme prematurity.

Our study consistently observed maternal hemodilution as a hallmark feature of Mirror Syndrome, which is consistent with prior studies.^7^ Maternal hemodilution characterized by low hematocrit distinguishes Mirror Syndrome from preeclampsia which is rather associated with hemoconcentration or isolated fetal hydrops with normal hematocrit.^6,9^ As expected, we also observed a rapid maternal weight gain, hypertension and hypoalbuminuria which aligns with prior reports.^6^

There are limited data comparing the effect of potential therapeutic interventions for hydrops on Mirror Syndrome outcomes, such as selective fetoscopic laser for twin-twin transfusion syndrome or fetal blood transfusion for anemia on the resolution of Mirror Syndrome. The systematic review by Allarakia et.al showed promising results of different treatment modalities including insertion of a peritoneal-amniotic shunt or thoraco-amniotic shunt, and aortic valve dilation and induction of labor to improved survival of infants diagnosed with hydrops.^6^ In our study, fetal and neonatal outcomes were not significantly improved in the two cases where therapeutic interventions were utilized, and maternal symptoms only improved following delivery.

The strengths of our study include a consecutive sample of all cases of fetal hydrops within the study centres alongside detailed information on associated factors and the clinical course of the condition. Limitations include the retrospective nature of the study and a small sample size, making it difficult to comment on the prognostic value of identified associated factors.

In conclusion, this case series adds important data to the existing limited literature on Mirror Syndrome. We affirm that maternal hemodilution is an important diagnostic characteristic to differentiate from other similar entities and that delivery occurs soon after diagnosis in most instances. Maternal symptoms usually improve within several days after delivery, however fetal outcomes if associated with structural anomalies and extreme prematurity, are poor. Further awareness of this rare condition is important, particularly as there are some etiologies which may be amenable to fetal intervention which could potentially improve pregnancy outcomes. These areas require further investigation as we learn more regarding the pathophysiology, treatment options and perinatal outcomes for Mirror Syndrome.
